# Correlation between *In Vitro* Cytotoxicity and *In Vivo* Lethal Activity in Mice of Epsilon Toxin Mutants from *Clostridium perfringens*

**DOI:** 10.1371/journal.pone.0102417

**Published:** 2014-07-11

**Authors:** Jonatan Dorca-Arévalo, Serge Pauillac, Laura Díaz-Hidalgo, Mireia Martín-Satué, Michel R. Popoff, Juan Blasi

**Affiliations:** 1 Laboratory of Cellular and Molecular Neuroscience, Department of Pathology and Experimental Therapeutics, Universitat de Barcelona, L'Hospitalet de Llobregat, Barcelona, Spain; 2 IDIBELL-Bellvitge Biomedical Research Institute, L'Hospitalet de Llobregat, Barcelona, Spain; 3 Institut Pasteur, Unité des Bactéries anaérobies et Toxines, Paris, France; Monash University, Australia

## Abstract

Epsilon toxin (Etx) from *Clostridium perfringens* is a pore-forming protein with a lethal effect on livestock, producing severe enterotoxemia characterized by general edema and neurological alterations. Site-specific mutations of the toxin are valuable tools to study the cellular and molecular mechanism of the toxin activity. In particular, mutants with paired cysteine substitutions that affect the membrane insertion domain behaved as dominant-negative inhibitors of toxin activity in MDCK cells. We produced similar mutants, together with a well-known non-toxic mutant (Etx-H106P), as green fluorescent protein (GFP) fusion proteins to perform *in vivo* studies in an acutely intoxicated mouse model. The mutant (GFP-Etx-I51C/A114C) had a lethal effect with generalized edema, and accumulated in the brain parenchyma due to its ability to cross the blood-brain barrier (BBB). In the renal system, this mutant had a cytotoxic effect on distal tubule epithelial cells. The other mutants studied (GFP-Etx-V56C/F118C and GFP-Etx-H106P) did not have a lethal effect or cross the BBB, and failed to induce a cytotoxic effect on renal epithelial cells. These data suggest a direct correlation between the lethal effect of the toxin, with its cytotoxic effect on the kidney distal tubule cells, and the ability to cross the BBB.

## Introduction

Epsilon toxin (Etx) is the most potent clostridial toxin after botulinum and tetanus neurotoxins. It is produced by types B and D strains of the anaerobic bacterium *Clostridium perfringens* and primarily affects livestock in the form of rapidly fatal enterotoxemias, resulting in heavy economic losses [1]. The lethal effect of Etx has been associated with general edema and neurological alterations, leading to a glutamate-mediated excitotoxic effect and neuronal death [2], [3], [4], [5]. In addition, Etx also affects the renal system, producing congestion, interstitial hemorrhage and cytotoxicity of epithelial distal tubule cells [6], [7], [8]. However, the individual implication of these effects on the lethal activity of Etx is still a matter of controversy [6], [7], [8], [9]. There is no direct evidence of human infections by *Clostridium perfringens* type B and D, although the very low LD50 of Etx in all experimental species tested to date suggest that this toxin might be toxic to humans [10], [11], [12]. Recently, it has been postulated that Etx could contribute to nascent Multiple Sclerosis lesion formation [13]

Etx is produced as a less toxic precursor molecule (proEtx), which is activated upon proteolytic cleavage of amino- and carboxy-terminal peptides [14]. Non-active proEtx presumably binds to the same cell surface receptors as the fully active molecule and can prevent its binding and further toxicity [7], [15], [16].

The most Etx-sensitive cell line known to date is the Madin-Darby canine kidney (MDCK) cell line of epithelial origin from the distal convoluted tubule [17]. The cytotoxic effect of Etx has been also demonstrated in a series of cell lines and cultures of renal origin: in a highly differentiated murine renal cortical collecting duct principal cell line, mpkCCD_cl4_ [18], in the human renal adenocarcinoma cell line ACHN [19], in the human renal leiomyoblastoma cell line G-402 [20], and in primary cultures of human renal tubular epithelial cells (HRTEC) [21]. In addition to cellular models of renal origin, primary cultures of mice cerebellar cortex granule cells are also targeted and affected by Etx [22]. Evidence based on studies using MDCK cells indicates that Etx is a pore-forming protein that causes ion homeostasis deregulation and cell death [23]. The toxin follows a series of steps in the cell intoxication pathway: binding to a specific receptor on the surface of host cells; oligomerization, forming a heptameric pre-pore complex; and insertion into the plasma membrane producing an active pore with the subsequent ionic deregulation and cell death [18], [22], . These three steps have been defined as decisive in the intoxication pathway of Etx in MDCK cells [23], [26]. However, the removal of cholesterol from mpkCCD_cl4_ cell membrane impairs Etx oligomerization and pore formation, but does not block cellular ATP release and cell necrosis, suggesting a Etx cytotoxic mechanism infdependent of pore formation [18].

Etx mutants are valuable molecular tools to study the cellular and molecular mechanisms of Etx activity [19], [28], [29], [30]. Recently, two dominant-negative inhibitors of *Clostridium perfringens* Etx have been designed: Etx-I51C/A114C and Etx-V56C/F118C. These dominant-negative mutant proteins have paired cysteine substitutions at locations predicted to form a disulfide bond that would impede toxin insertion into the plasma membrane and the subsequent pore formation. Cytotoxic activity can be reconstituted in both mutant proteins by incubation with dithiothreitol (DTT), a reducing agent. When equimolar mixtures of wild-type Etx and mutant proteins were added to MDCK cells, both mutant proteins prevented wild-type Etx from forming stable oligomeric complexes in the cells, which is a step required for pore formation and cytotoxicity [30].

However, *in vivo* studies have not been performed on the possible lethal effect of the paired cysteine mutants and their distribution in target organs (especially kidneys and brain). Mice have been widely used to study the effect of Etx and provide a useful model for laboratory controlled intoxication studies [31], [32], [33], [34], [35]. As in sheep and other naturally sensitive species, the toxin can cross the BBB in mice and enter the brain parenchyma [36].

We used Etx fusion proteins (wild type and mutants) with the green-fluorescence protein (GFP-Etx, GFP-Etx-I51C/A114C and GFP-Etx-V56C/F118C), and injected it intravenously in mice to study their possible lethal activity and their distribution in the target organs by direct fluorescence. A well-established non-toxic mutant of Etx which induces protective immunity in mice [29] was also used as a fusion protein (GFP-Etx-H106P) and compared to Etx wild type and mutants. The results demonstrate that ability to cross the blood-brain barrier (BBB) is related to epithelial damage to the renal distal tubules and also with lethal effects.

## Materials and Methods

### Ethics statement

Male OF1 Swiss mice were housed under conventional conditions in climatized and pathogen-free rooms with free access to standard pelleted food and tap water. The experiments were performed according to EU guidelines for animal experimentation and the protocols used were approved by the University of Barcelona's Committee on the Ethics of Animal Experiments (Ref. Number: 350/12). The experiments were carried out in the animal research facility of the University of Barcelona (Campus de Bellvitge) in animal preparation rooms with the required equipment for isoflurane anesthesia and animal preparation. All efforts were made to minimize suffering.

### Cloning, expression and purification of GFP-Etx wild type and mutants

Etx wild type and mutants were produced as fusion proteins with GFP as previously described [36], starting at amino acid A47 [37], which lacks the N-terminal peptide, but not the C-terminal peptide that was removed by trypsin digestion to produce active toxin when required. This treatment activates Etx but does not digest GFP. The nomenclature for the mutants follows that previously used by Oyston et al. (1998) and Pelish and McClain (2009). The mutations Etx-H106P [29], Etx-I51C/A114C and Etx-V56C/F118C [30] were introduced into the cloned *etxB* gene using the QuikChange multi-site-directed mutagenesis kit (Stratagene), following the manufacturer's instructions and using the primers: (5′-AACTGCAACTACTACTCCTACTGTGGGAACTTCGA-3′) for Etx-H106P, (5′-AGGAAATGATTTTTATTGTAATAATCCTAAAGTTG-3′/5′-GGGAACTTCGATACAATGTACTGCTAAGTTTACTG-3′) for Etx-I51C/A114C, and (5′-TATTAATAATCCTAAATGTGAATTAGATGGAGAAC-3′/5′-ACAAGCAACTGCTAAGTGTACTGTTCCTTTTAATG-3′) for Etx-V56C/F118C.

Wild type and mutated DNA were cloned into the pGEX-4T-1 vector (GE Healthcare Life Sciences) containing the EGFP coding sequence to produce the corresponding recombinant fusion proteins, as previously described [36]. All constructs were sequenced to confirm that the mutations were introduced into the proper positions and no errors were present. The expression of the different proteins was induced overnight in the presence of 1 mM isopropyl beta-D-thiogalactopyranoside (IPTG) at room temperature (RT), in 250 ml of LB medium containing 50 µg/ml of ampicillin. Cells were pelleted and resuspended in ice cold phosphate buffer (PB) 20 mM pH 7.5 with NaCl 250 mM, sonicated and centrifuged at 12,000×g for 20 min. The resultant supernatant was incubated with Glutathione Sepharose 4B beads (GE Healthcare Life Sciences) for 1 h at 4°C. Finally, the recombinant proteins were eluted by thrombin cleavage in 20 mM PB, pH 7.5 containing 250 mM NaCl and 2.5 mM CaCl_2_. When required, wild type and mutated GFP-proEtx were fully activated by incubation with trypsin-coated agarose beads for 30 min at RT (Sigma–Aldrich, Madrid, Spain). Protein concentration was determined by the method of Bradford [38], using bovine serum albumin (BSA) as standard. The purity of the recombinant proteins was checked by SDS-PAGE and Coomassie Blue staining.

### Confocal microscopy on MDCK cells and mouse tissue cryosections

MDCK cells (ATCC, CCL-34) were grown on coverslips to confluence in DMEM-F12 medium supplemented with L-Glutamine, 15 mM HEPES and antibiotics. Cells were washed three times with PBS, and fixed with 4% paraformaldehyde (PFA) for 12 min at RT. After washing with PBS, cells were blocked by adding PBS containing 20% normal goat serum (NGS) for 1 h at RT and incubated with 300 nM of GFP-proEtx or GFP-proEtx mutants in PBS containing 1% NGS for 1 h at RT. After 3 washes with PBS, the samples were stained with TO-PRO-3 (1∶1000 dilution, Molecular Probes, Invitrogen) for 7 min, washed again and mounted with an aqueous mounting medium (Fluoromount, SIGMA).

Incubation of MDCK cells and tissue samples with GFP-proEtx wild type and mutants was performed according to previous studies [6], [39]. Mouse kidney and brain samples were fixed immediately after extraction by immersion in 4% PFA for 12 h. Samples were cryoprotected by immersion in 30% sucrose, embedded in OCT medium and snap-frozen in precooled isobutanol. Cryostat sections of 10 µm were obtained, mounted onto poly-lysine coated microscope slides (Superfrost Plus, Thermo scientific), and stored at −20°C until used. Slides were examined in a Leica TCS-SL, spectral confocal microscope (CCiTUB, Biology Unit of Bellvitge Campus). The relative level of fluorescence for each condition, fusion proteins of GFP and Etx wild type and mutants, was measured using ImageJ software (http://rsbweb.nih.gov/ij/index.html).

### Cytotoxicity assay and protein complex formation in MDCK cells

MDCK cells were grown to 80% confluence in DMEM F-12 medium supplemented with 10% fetal bovine serum and antibiotics and mpkCCD_c14_ cells were grown in DMEM/Ham's F-12 (1∶1, v/v) containing 60 nM sodium selenate, 5 µg/ml transferrin, 2 mM glutamine, 50 nM dexamethasone, 1 nM triiodothyronine, 10 ng/ml epidermal growth factor, 5 µg/ml insulin, 20 mM D-glucose, 2% fetal calf serum, and 20 mM HEPES, pH 7.4.

The cytotoxic activity of wild type derivatives and mutated Etx was determined using three different assays: the propidium iodide influx assay [40] to check the efficiency of wild type activated Etx, the MTT assay [6], and the real-time xCELLigence impedance analysis [41]. The propidium iodide influx assay was used to characterize the cytotoxic activity of Etx fusion protein (with GFP) compared to wild type toxin, while the MTT assay was used to study the high dose-dependence of Etx mutants and the effect of reducing agent, as previously described [30]. Values were compared taking 0.5% of triton ×100 incubated cells as 100% mortality and cells incubated with GFP alone as 0% of cell mortality.

The xCELLigence System (ACEA) monitors the cellular events in real-time by measuring electrical impedance across microelectrodes on the bottom of each well. In these experiments, MDCK or mpkCCDc14 cells were seeded on E-plate 16 then treated with or without the 10 nM of each trypsin-treated ETX sample. The electrical impedance was measured every 15 min by RTCA-integrated software from the xCELLigence system and derived as a dimensionless parameter termed the cell index (CI), taking into account the biological status of the cells (cell number, adhesion, morphology and viability).

To analyze the formation of Etx protein complexes at the plasma membrane, MDCK cells were grown to confluence in 10 cm plastic petri dishes and incubated with wild type and GFP-Etx mutants, as described above.

After incubation, cells were washed once with ice-cold PBS supplemented with protease inhibitor cocktail (Sigma) and scraped off with a rubber policeman in 150 µl of the same buffer. Harvested cells were lysed by one cycle of freezing and thawing and homogenized by passage through a 29-gauge needle. Nuclei were removed by centrifugation at 900×g for 10 min at 4°C. The supernatant was centrifuged at 20,000×g for 30 min at 4°C and the pellet, which was considered the crude membrane fraction, was homogenized with ice-cold PBS supplemented with protease inhibitor cocktail, quantified and analyzed by Western blotting using anti-GFP rabbit polyclonal antibody.

### *In vivo* studies in a murine model

Male OF1 Swiss mice weighing 20 g were anaesthetized by administration of isoflurane before the intravenous injection of GFP-Etx, GFP-proEtx or GFP-Etx mutants at a final dose of 2.5 µg/g of body weight with 200 µg BSA-Alexa 647 in PBS containing 1% BSA. All animals injected with GFP-Etx and the mutant GFP-Etx-I51C/A114C died between 5 and 15 min after injection. No lethal effect was observed (up to 24 h post-injection) with either GFP-proEtx, GFP-Etx-H106P and GFP-Etx-V56C/F118C, and animals were routinely processed for histopathological analysis after 15 min of i.v. injection. Each experimental procedure was performed three times with duplicates for each group (toxin mutant). To minimize animal suffering, mice were anesthetized before i.v. injections and maintained under anesthesia until the end of the experiment. Animals died as a direct result of the intervention (Etx effect) or were sacrificed by cervical dislocation (controls and non-toxic Etx mutants), in all cases under deep anesthesia.

Brains and kidneys were removed, fixed by immersion in 4% PFA for 12 h, immersed in 30% sucrose, frozen in isobutanol and cut into 10 µm sections in a cryostat as detailed above. Some sections were immediately mounted on slides using Immuno-Fluore Mounting Medium (ICN Biomedicals; Costa Mesa, CA) and examined under a Leica TCS-SL spectral confocal microscope (CCiTUB, Biology Unit of the Bellvitge Campus) for direct binding of GFP-Etx to brain and kidney samples. Alternatively, sections were processed for immunofluorescence and confocal microscopy. Non-specific binding was blocked by incubating the sections in PBS containing 20% NGS and 0.2% gelatin for 1 h at RT. The primary polyclonal anti-glial fibrillary acidic protein (GFAP) antibody (Sigma, 1/500) was added and sections were incubated O/N at 4°C in PBS containing 1% NGS. Alexa-546 labelled goat anti-rabbit antibody (Dako, 1/500) was used as a secondary antibody, and incubated for 1 h before washing and mounting the slides for confocal microscopy observation.

## Results

### Expression and cytotoxic characterization of recombinant GFP-proEtx mutants

**Figure 1:**
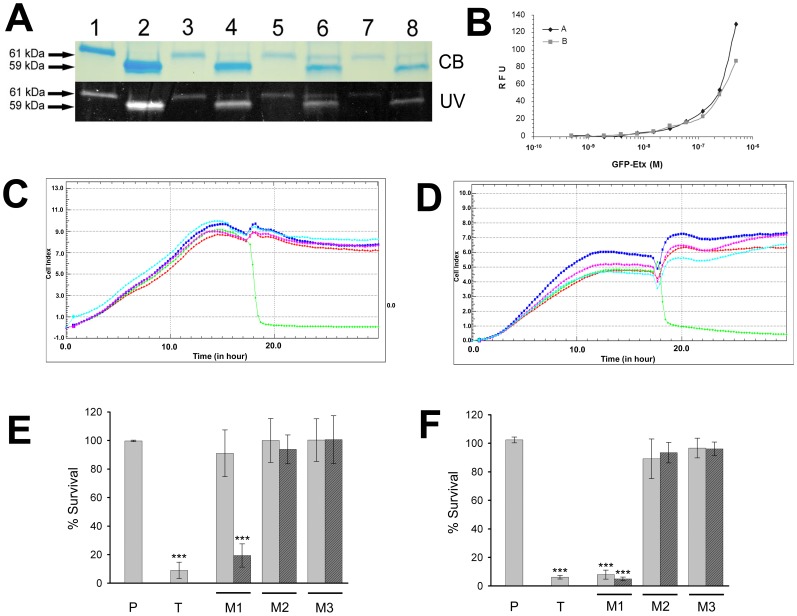
Characterization and cytotoxic effect of GFP-Etx-mutants. A) GFP-proEtx (1), GFP-proEtx-I51C/A114C (3), GFP-proEtx-V56C/A118C (5) and GFP-proEtx-H106P (7) were activated by trypsin treatment to produce GFP-Etx (2), GFP-Etx-I51C/A114C (4), GFP-Etx-V56C/A118C (6) and GFP-Etx-H106P (8). Samples were analyzed by SDS-PAGE followed by Coomassie Blue staining (CB) and direct visualization on a UV transilluminator (UV). Note the shift in the molecular weight after trypsin activation. B) The dose-response cytotoxic effect induced by GST-GFP-ETX wild type (gray square) was measured by the PI exclusion test and compared with native Etx (black rhombus). Results are expressed as relative fluorescence units (RFU). C and D) Dynamic monitoring of toxin effects on MDCK (B) or mpkCCD_c14_ cells (C) using the xCELLigence System. Cells seeded in E-Plate16 were incubated with 10 nM of trypsin-treated GFP-Etx (green), GFP-Etx-I51C/A114C (blue), GFP-Etx-V56C/A118C (purple) or GFP-Etx-H106P (cyan). All the Etx mutant fusion protein-treated cells behaved similarly to the untreated control (red). Each curve is an average of n = 4. Error bars were omitted for reasons of clarity, but never exceeded 10% of the average value. E) MDCK cells were incubated with 6.5 nM of GFP-proEtx (P), GFP-Etx (T), GFP-Etx-I51C/A114C (M1), GFP-Etx-V56C/A118C (M2) and GFP-Etx-H106P (M3) in the presence (dark grey bars) or absence (light grey bar) of 3 mM DTT. GFP-proEtx and GFP-Etx-H106P were used as a negative non-toxic control, whereas GFP-Etx was used as a positive control. Student's t-test indicates significant differences (***p<0.001) for cells incubated either with GFP-Etx or GFP-Etx-I51C/A114C after DTT treatment. F) MDCK cells were incubated with 50 nM of GFP-proEtx (P), GFP-Etx (T), GFP-Etx-I51C/A114C (M1), GFP-Etx-V56C/A118C (M2) and GFP-Etx-H106P (M3) in the presence (dark grey bars) or absence (light grey bar) of 3 mM DTT. GFP-proEtx and GFP-Etx-H106P were used as a negative non-toxic control, whereas GFP-Etx was used as a positive control. Student's t-test indicates significant differences (***p<0.001) for cells incubated either with GFP-Etx or GFP-Etx-I51C/A114C, treated with or without DTT.

GFP-proEtx, wild type (GFP-Etx) and GFP-Etx mutants were cloned and successfully expressed in *E. coli* as GST fusion proteins. After purification and SDS-PAGE electrophoresis analysis, fluorescent bands due to the GFP moiety of the fusion protein were observed in a UV transilluminator. The fluorescent bands corresponded to the Coomassie Blue stained bands of the expected molecular size for GFP-proEtx (Figure 1A). The cytotoxic activity of trypsin-activated tagged GFP-Etx was compared to native Etx by the propidium iodide influx assay (Figure 1B). Additionally, to check the cytotoxic activity of Etx recombinant proteins (wild type and mutants) a real-time xCELLigence impedance analysis was performed on MDCK (Figure 1C) and mpkCDD_c14_ (Figure 1D) cell lines. GFP-Etx mutants showed no cytotoxic activity at a concentration of 10 nM compared to the wild type GFP-Etx toxin (Figure 1C and D). In addition, to check whether a reducing agent (DTT) restores the cytotoxic activity of the mutants with paired cysteine substitutions, MTT assays were performed on MDCK cells exposed to 6.5 nM of each of the GFP-Etx mutants (treated with or without DTT) and the results were compared to those obtained with GFP-proEtx and GFP-Etx (Figure 1E).

At this concentration, the mutant GFP-Etx-I51C/A114C did not show any cytotoxic effect on MDCK cells unless it was preincubated with DTT (Figure 1E). However, the GFP-Etx-V56C/F118C and GFP-Etx-H106P mutants did not even have a cytotoxic effect on MDCK cells when preincubated with DTT (Figure 1E). At higher concentrations of the GFP-Etx mutants, starting from 50 nM, mutant GFP-Etx-I51C/A114C had an evident cytotoxic effect on MDCK cells, even in the absence of DTT (Figure 1F). This effect contrasted with that of mutants GFP-Etx-V56C/F118C and GFP-Etx-H106P, which did not show any cytotoxic activity at these concentrations, even in the presence of a reducing agent (DTT), similarly to the GFP-proEtx effect.

### Binding of GFP-proEtx mutants to MDCK cells, brain and kidney sections from mouse

Previous studies demonstrated that GFP-Etx binds with high affinity to the plasma membrane of MDCK cells [39]. Moreover, incubations of mouse brain and kidney sections with GFP-Etx revealed that it bound to myelinic structures and to the distal and convoluted tubules respectively [6], [16], [39].

To study and compare the binding properties of GFP-proEtx wild type and mutants, MDCK cells and mouse brain and kidney sections were incubated with the GFP-proEtx mutants, GFP-proEtx-I51C/A114C, GFP-proEtx-V56C/F118C and GFP-proEtx-H106P.

**Figure 2:**
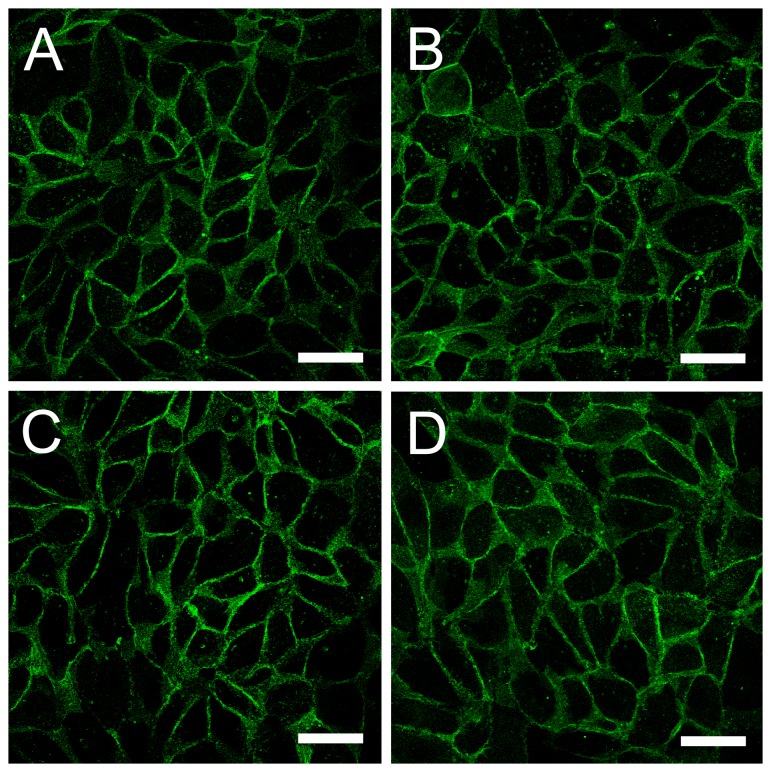
Binding of GFP-Etx-mutants to MDCK cells. Confocal microscopy images of MDCK cells showing the binding of GFP-Etx-mutants to the plasma membrane (green). GFP-proEtx (A), GFP-proEtx-I51C/A114C (B), GFP-proEtx-V56C/A118C (C) and GFP-proEtx-H106P (D). Scale bar: 40 µm.

**Figure 3:**
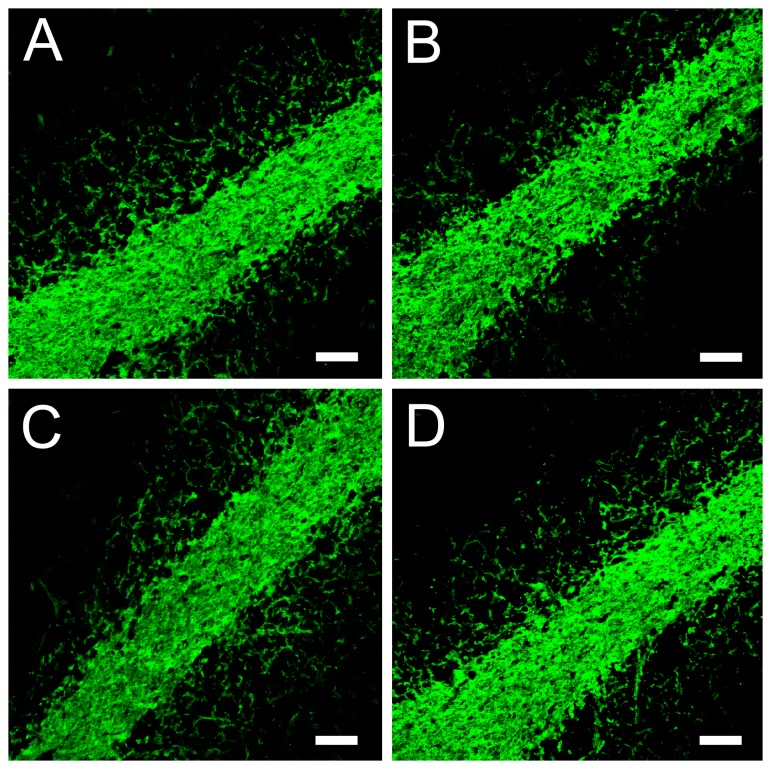
Binding of GFP-Etx-mutants to myelinic structures from mouse cerebellum. Confocal microscopy images showing the binding of GFP-Etx-mutants to myelinic structures from the mouse cerebellum. GFP-proEtx (A), GFP-proEtx-I51C/A114C (B), GFP-proEtx-V56C/A118C (C) and GFP-proEtx-H106P (D). Scale bar corresponds to 40 µm.

**Figure 4:**
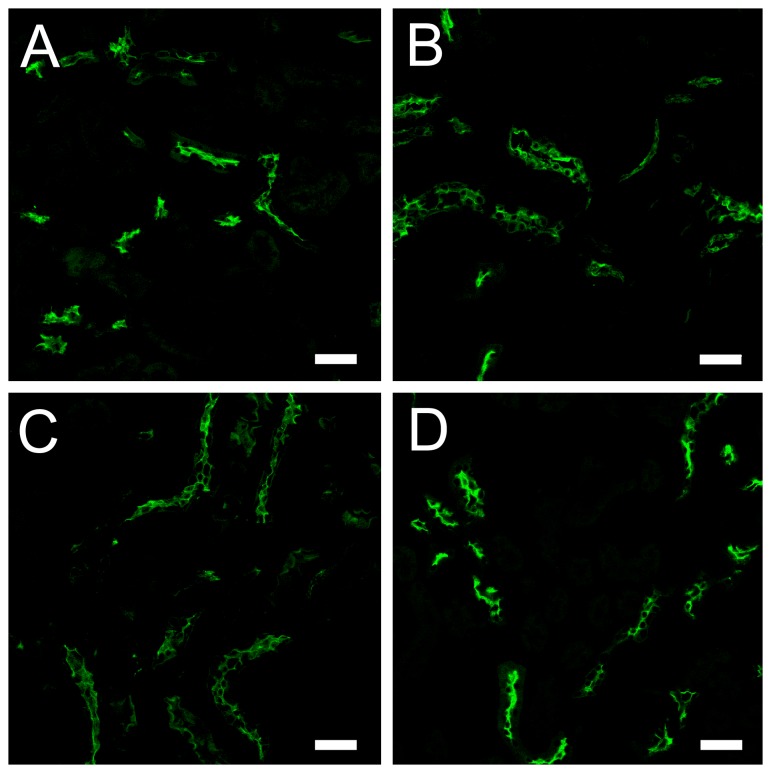
Binding of GFP-Etx-mutants to distal and collecting tubules from mouse kidney. Confocal microscopy images showing the binding of GFP-Etx-mutants to distal and collecting tubules from the mouse kidney. GFP-proEtx (A), GFP-proEtx-I51C/A114C (B), GFP-proEtx-V56C/A118C (C) and GFP-proEtx-H106P (D). Scale bar corresponds to 40 µm.

Similarly to wild type proEtx, incubations of MDCK cells with the proEtx mutants revealed binding to the plasma cell membrane (Figure 2) with no significant differences in fluorescence intensity compared to wild type (not shown). Incubations with brain and kidney sections revealed binding of proEtx mutants to myelinic structures and distal tubules, respectively (Figure 3 and Figure 4). Therefore, all of the GFP-Etx mutants tested showed a similar binding pattern to GFP-Etx, which clearly suggests that binding to target cells and tissues is not affected in any of the mutants.

**Figure 5:**
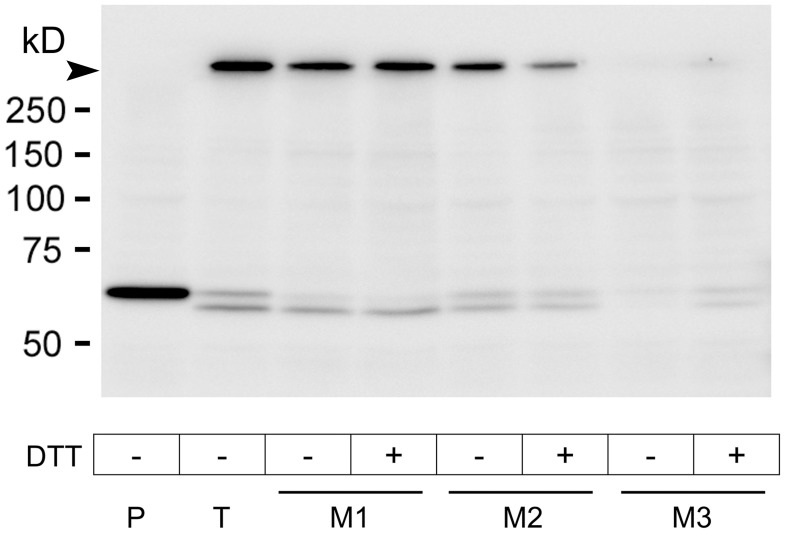
Oligomeric complex formation in MDCK cells treated with GFP-Etx and GFP-Etx mutants. MDCK cells were incubated with GFP-proEtx (P), GFP-Etx (T), GFP-Etx-I51C/A114C (M1), GFP-Etx-V56C/A118C (M2) and GFP-Etx-H106P (M3) at 6.5 nM for 30 min at 37°C. GFP-Etx mutants were previously treated (+) or not (−) with 3 mM of DTT for 1 h at 45°C. The cytotoxic effect was found only after incubations with GFP-Etx and GFP-Etx-I51C/A114C treated with DTT (see Figure 2). Western blot analysis revealed the presence of protein complex formation (arrowhead) in all cases except for GFP-proEtx.

To analyze the formation of toxin oligomers by wild type Etx compared to Etx mutants in the plasma membrane, MDCK cells were incubated with wild type or Etx mutants, and the presence of Etx protein complexes was analyzed by Western blot assay (Figure 5).

### *In vivo* experimental mouse model

In a previous study, fluorescence microscopy analysis of cryostat sections from various organs of toxin-injected mice demonstrated specific, displaceable binding of GFP-Etx to blood vessels of brain and to distal tubules of kidneys, and unspecific accumulation to proximal tubules [36]. Similarly, immunofluorescence of brain sections also identified toxin binding sites in defined regions of mouse cerebellar cortex [22]. We therefore were interested in determining the correlation between the observed cytotoxic activity on MDCK cells and the distribution of wild-type and mutant Etx in target organs (especially in brain) and the abilities of the toxins to cross the BBB.

We first evaluated the mutant toxins for lethal effects *in vivo*. Both GFP-Etx and the mutant GFP-Etx-I51C/A114C were observed to be lethal between 5 and 15 minutes after an i.v. injection in the mouse tail vein. The mutants GFP-Etx-V56C/F118C and GFP-Etx-H106P did not have a lethal effect on injected animals, at least in the first 24 h after i.v. injection, which was the maximum post-injection time allowed before processing the organs for histopathological analysis. Therefore, in most cases, when GFP-Etx-mutants showed no lethal effect, animals were processed for histopathological analysis and fluorescence distribution of the injected GFP-Etx forms, no more than 15 min after injection.

**Figure 6:**
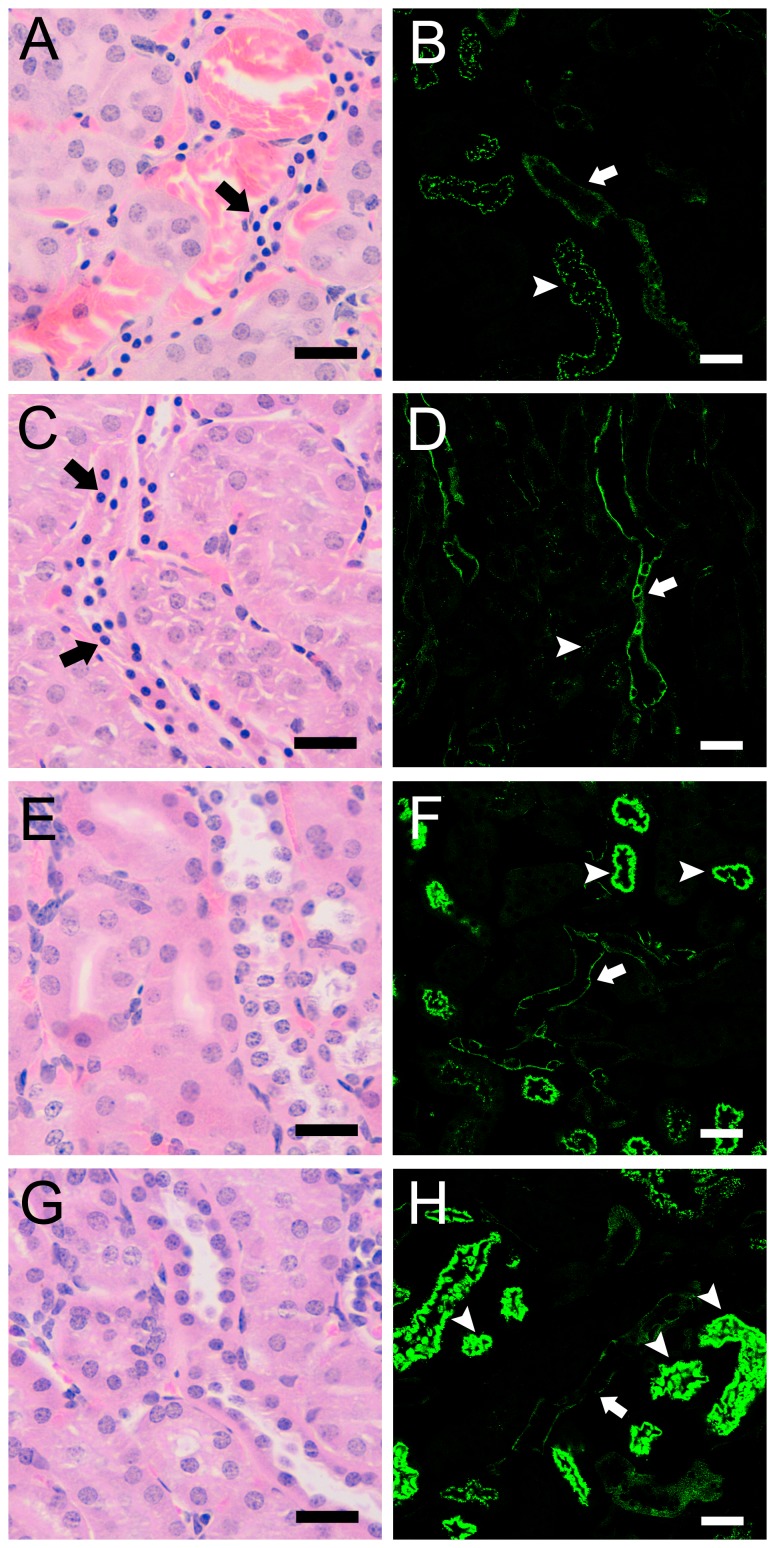
Effect of GFP-Etx and GFP-Etx-mutants on distal and collecting renal tubules after i.v. injection. Hematoxilin-eosin staining (A, C, E, and G) and confocal microscopy images (B, D, F and H) of kidney sections from mice injected with GFP-Etx (A and B), GFP-Etx-I51C/A114C (C and D), GFP-Etx-V56C/A118C (E and F) and GFP-Etx-H106P (G and H). All the Etx fusion proteins bind specifically to the epithelial cells of distal and collecting renal tubules (white arrows, in green) and unspecifically to the luminal part of proximal tubules (white arrowheads, in green). Only the GFP-Etx and GFP-Etx-I51C/A114C mutants produce a cytotoxic effect on epithelial cells from distal and collecting tubules (black arrows, A and C). Scale bar correspond to 25 µm in A, C, E and G and 40 µm in B, D, F and H.

Kidneys from GFP-Etx and GFP-Etx-I51C/A114C injected mice revealed congested and hemorrhagic medullae compared to animals injected with either GFP-Etx-V56C/F118C or GFP-Etx-H106P (Figure 6E–H). Confocal images corresponding to the fluorescence of wild type and mutated fusion proteins were obtained from the epithelial cells of distal tubules and also in the apical domain of epithelial proximal tubules. However, only mice that received injections containing GFP-Etx and GFP-Etx-I51C/A114C showed pyknotic nuclei in epithelial cells from distal tubules (Figure 6A, C) similarly to previous observations in Etx intoxicated animals [6], which suggests a cytotoxic effect of both molecules on these cells. However, neither apoptotic nor necrotic processes could be defined as a possible mechanism of cell death after the short period of time between Etx injection and animal death. Conversely, no alterations were observed in kidneys from animals injected with GFP-Etx-V56C/F118C or GFP-Etx-H106P mutants (Figure 6E,G).

**Figure 7:**
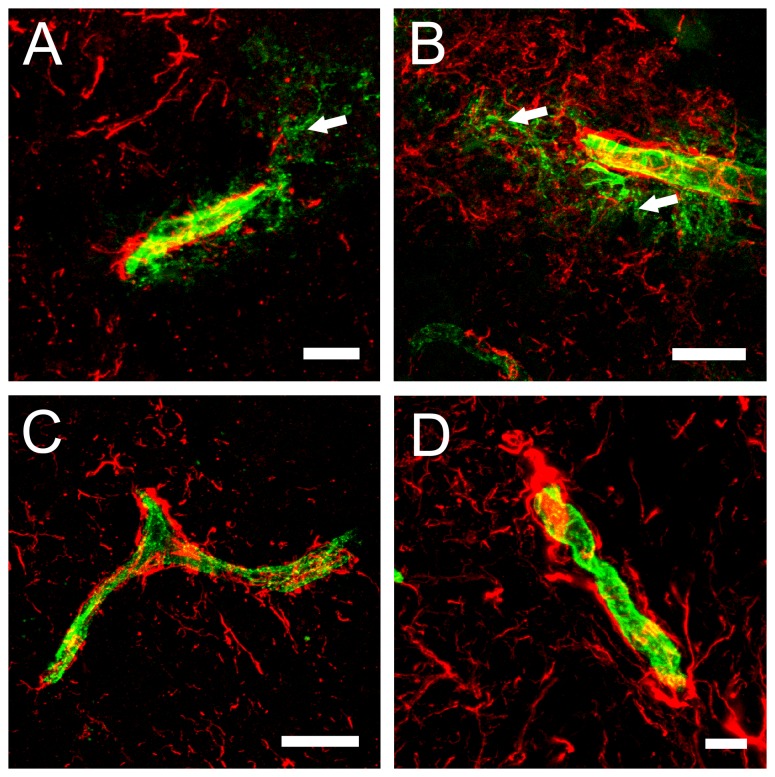
Brain sections from injected mouse with GFP-Etx and GFP-Etx-mutants. Confocal microscopy images of brain sections from mice injected with GFP-Etx (A), GFP-Etx-I51C/A114C (B), GFP-Etx-V56C/A118C (C) and GFP-Etx-H106P (D), and immunostained with anti-GFAP (red). Only GFP-Etx (A) and GFP-Etx-I51C/A114C (B), could cross the blood-brain barrier and get into the brain parenchyma (white arrows). Scale bar corresponds to 40 µm.

Confocal images from brain sections revealed the accumulation of GFP-Etx (Figure 7A), GFP-Etx-I51C/A114C (Figure 7B), GFP-Etx-V56C/F118C (Figure 7C) and GFP-Etx-H106P (Figure 7D) on the luminal surface of the vascular endothelium in all brain areas. In the case of GFP-Etx and GFP-Etx-I51C/A114C, fluorescence was also detected in the brain parenchyma surrounding some blood vessels (Figure 7A–B, arrows), which suggests that GFP-Etx and GFP-Etx-I51C/A114C, but not GFP-Etx-V56C/F118C and GFP-Etx-H106P, could cross the BBB. In addition, a well-documented vasogenic edema, especially in brain microvasculature, that was produced by Etx (reviewed by Finnie 2004) was detected after GFP-Etx and GFP-Etx-I51C/A114C injection (data not shown).

**Figure 8:**
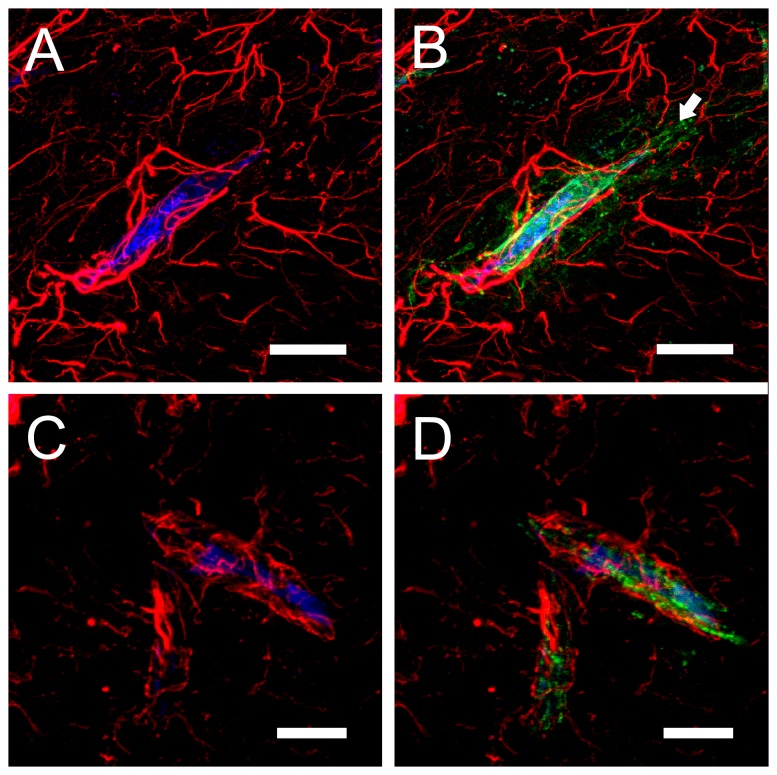
Brain sections from mouse co-injected with Alexa 647-labelled BSA and GFP-Etx-I51C/A114C or GFP-Etx-V56C/A118C. Mice were i.v. injected with GFP-Etx-I51C/A114C (A–B) or GFP-Etx-V56C/A118C (C–D) in combination with Alexa 647-labelled BSA. Brains were processed for cryostat sectioning and examined under a confocal microscope. Green fluorescence corresponds to GFP-Etx-I51C/A114C (B) or GFP-Etx-V56C/A118C (D) and blue fluorescence corresponds to BSA (A–D). Red fluorescence corresponds to GFAP labeling (astrocytic processes and perivascular end-feet, A–D). Notice that in GFP-Etx-V56C/A118C-injected mice, both BSA and the GFP-Etx mutant remained confined in the blood vessel's lumen surrounded by the astrocyte's perivascular end-feet (C and D), while in GFP-Etx-I51C/A114C-injected mice, BSA remained in the lumen but the GFP-Etx mutant leaked out of the vessel, beyond astrocytic processes (A and B, arrows). Scale bars correspond to 20 µm (A and B) and 16 µm (C and D).

To check the integrity of the BBB in injected animals, we performed experiments in which BSA labeled with Alexa-647 was co-injected with the same doses of toxin or mutants indicated above. In GFP-Etx-V56C/F118C (Figure 8 C, D) and GFP-Etx-H106P (not shown) injected animals, green fluorescence was associated with endothelia, as seen above, while the fluorescence corresponding to Alexa-647-BSA (in blue), was detected inside the blood vessels (Figure 8 C, D). In the case of GFP-Etx (not shown) and GFP-Etx-I51C/A114C injected animals (Figure 8 A, B), Alexa-647-BSA was confined to the vessels and surrounded by the astrocyte's perivascular end-feet, while GFP-Etx (not shown) and GFP-Etx-I51C/A114C (Figure 8A, B, arrow) were clearly found deep in the brain parenchyma. These results indicate that both GFP-Etx and GFP-Etx-I51C/A114C not only permeabilize the vascular endothelium, but also cross the BBB and accumulate in the brain parenchyma.

## Discussion

The present study shows that two Etx mutants designed to elude the pore formation step in the toxin's mechanism of action have a differential *in vivo* effect but maintaining the binding and oligomerization abilities on target cells. This is an important finding as the cytotoxic activity of Etx has been basically associated with its ability to oligomerize and form pores in the cell membrane of target cells.

Etx from *Clostridium perfringens* crosses the blood-brain barrier (BBB) and gets into the brain parenchyma [36], [42], [43], [44], [45], [46], [47] where it produces neuronal damage by acting on the glutamatergic system and evoking excessive release of glutamate [4]. Moreover, it specifically binds to MDCK cells [48] and to the epithelium of distal tubules from the kidneys, where it produces severe kidney alterations [6], [7]. We have previously demonstrated that recombinant GFP-Etx is a convenient tool to reproduce the same *in vivo* effects as native Etx [6], [36].

Two of the mutants of *Clostridium perfringens* Etx used in this study, Etx-I51C/A114C and Etx-V56C/F118C [30], have paired cysteine substitutions that are predicted to form a disulfide bond that would prevent the unfolding of the loop involved in Etx insertion into the cell membrane, a situation that may be reverted after treatment with a reducing agent such as DTT. Etx wild type and mutants were generated as fusion proteins with GFP, to further detect the protein under fluorescence and confocal microscopy. The particular behavior of each mutant was checked first on the basis of their binding and cytotoxic effect on MDCK cells, and afterwards by i.v. injections in mice to analyze the impact on kidneys and brain as the main target organs. These effects were compared with those observed for the GFP-Etx wild type and the non-lethal GFP-Etx-H106P mutant [29]. This last mutant does not have paired cysteine substitutions inserted or disulfide bonds, but maintains the binding ability of the wild type molecule and is completely non-toxic [29].

Confocal images of MDCK cells incubated with the GFP-Etx wild type and mutants revealed that all the mutants retain the binding ability of the native Etx to the plasma membrane. Moreover, incubation on mouse kidneys and brain sections showed that all Etx mutants used in this study bound to distal tubules and myelinic structures, respectively, similarly to wild type GFP-Etx. These results demonstrate that the paired cysteine substitutions in the mutated Etx do not affect the binding domain of the toxin, at least not functionally. In accordance with a previous report [30], MTT assays on MDCK cells revealed that all the Etx mutants were non-cytotoxic at 6.5 nM, possibly due to the disulfide bonds that would hamper insertion of the toxin in the plasma membrane. The effect of disulfide bonds was evident after treatment of the mutant GFP-Etx-I51C/A114C with DTT, which activated the mutant to the GFP-Etx wild type cytotoxic levels. However, DTT did not exert a significant effect on the mutant GFP-Etx-V56C/F118C nor, as expected, on wild type and the GFP-Etx-H106P mutant.

Curiously, MTT assays on MDCK cells incubated with GFP-Etx mutants at 50 nM revealed a cytotoxic effect of the GFP-Etx-I51C/A114C mutant even in the absence of DTT. However, the GFP-Etx-V56C/F118C and GFP-Etx-H106P mutants did not show any cytotoxic activity, even in the presence of DTT. Although the GFP-Etx-V56C/F118C mutant remains non-toxic even at high concentrations and in the presence of reducing agents, it retains the ability to form protein oligomers, which suggests that it is possible to dissociate the oligomerization step from the subsequent pore-forming activity. This discrepancy in the behavior of the paired cysteine Etx mutants could be explained by the differential stability of disulfide bonds, which may be less stable in the GFP-Etx-I51C/A114C mutant than in GFP-Etx-V56C/F118C. It is also possible that the mutated amino acids V56 or F118, or both, are essential for the full expression of Etx cytotoxic activity. The ability to form protein oligomers is almost completely abolished in the Etx-H106P mutant, which suggests that the loss of a cytotoxic, lethal effect is due to this mutant's incapacity to form the protein complex in the cell membrane previous to the pore formation. Moreover, the proline introduced in position 106 would be responsible for this loss of activity, since substitution of histidine-106 for serine or alanine did not produce this effect [29]. The results shown here with GFP-Etx-I51C/A114C and GFP-Etx-V56C/F118C contrast with an earlier study [30] in which mutant GFP-Etx-V56C/F118C had cytotoxic effects on MDCK cells following DTT treatment. The difference in the results between both studies could be due to divergences in the experimental approaches that were used. These divergences include the cell viability assay used, the time of incubation of MDCK cells with Etx (16 h in the former study compared to 30 min in the present study), the cell culture conditions (culture media, plates and cell density) and the recombinant protein purification protocols. One or more of these variables may account for the differences found between both studies, and deciphering these variables would give an important clue to the mechanism of action of Etx in the cytotoxic process.

The proposed model of cytotoxic action of Etx on MDCK cells starts with its binding to the plasma membrane, followed by the formation of complexes in the plasma membrane and formation of a pre-pore with subsequent insertion into the membrane to form a functional pore, which would lead to cell death [23]. Additionally, evidence on the mechanism of action of Etx in the cell line of collecting renal tubules mpkCCD_c14_ [18], indicate that pore formation is not the only cause of cell death. Accordingly, we cannot exclude a cytotoxic mechanism of Etx on epithelial cells from distal tubules, which would not include the formation of a membrane pore.

Previous *in vivo* studies performed by intravenous injection of GFP-Etx in mice reproduced the proteinaceous edema and the cytotoxic activity on the epithelial cells of renal distal tubules induced by Etx [36]. Moreover, co-injection of GFP-Etx and fluorescent-labeled BSA confirm that only GFP-Etx was able to bind and cross the endothelium, and distribute far beyond the boundaries within which BSA was confined [36]. Similar results, compared to GFP-Etx, were observed in mice injected with the mutant GFP-Etx-I51C/A114C, even when it was not previously treated with DTT, and suggesting a glutamatergic effect, similar to Etx wild type. Furthermore, in order to determine whether the mouse serum contained a possible agent responsible for GFP-Etx-I51C/A114C activation, MDCK cells were incubated with mouse serum together with the GFP-Etx mutants at 6.5 nM, and no cytotoxicity was observed (data not shown). This indicates that the mouse serum alone is not responsible for possible activation of the GFP-Etx-I51C/A114C. These data suggest that the mouse toxicity found after intravenous injection of GFP-Etx-I51C/A114C may be due to the high dose administered, which would be in accordance with our results in MDCK cells incubated with the GFP-Etx mutant at 50 nM or above. In contrast, the injected GFP-Etx-V56C/F118C and GFP-Etx-H106P mutants did not cross the BBB, produce a cytotoxic effect on epithelial cells from distal tubules, or have a lethal effect. These results are also in accordance with those found in MDCK cells where the Etx-V56C/F118C and Etx-H106P mutants were not cytotoxic at any concentration used, which strongly suggests that the lethal effect of Etx is associated with its ability to cross the BBB and the cytotoxic effect on renal tubules.

All together, the present report may help to understand the toxic mechanism of one of the most potent natural toxins and provide new clues to design molecules with therapeutic or preventive capacity for Etx intoxication, by dissociating the pore-forming activity from the oligomerization step in the regular sequence of cell intoxication: binding, oligomeritzation, pore formation, and cell death.
